# Microstructural and Performance Analysis of TP304H/T22 Dissimilar Steel Welded Joints

**DOI:** 10.3390/ma16124474

**Published:** 2023-06-20

**Authors:** Jian Sun, Tong Wang, Fuguang Liu, Zhoubo Zhang, Yunhui Chen, He Lin, Hui Liu, Xiaohui Zhao, Xiaole Cheng

**Affiliations:** 1 School of Mechanical and Electrical Engineering, Xi’an Polytechnic University, Xi’an 710048, China; 2 Xi’an Key Laboratory of Modern Intelligent Textile Equipment, Xi’an 710048, China; 3 Xi’an Thermal Power Research Institute Co., Ltd., Xi’an 710054, China

**Keywords:** dissimilar welded joints, microstructure, hardness, tensile properties

## Abstract

In the power plant boiler industry, dissimilar steel welding is widely used in the connection of thermal power generation units. As an important component of the unit, research on the organizational properties of dissimilar steel welded joints has significant guidance for the life design of the joint. For the long-term service state of TP304H/T22 dissimilar steel welded joints, the microstructure’s morphological evolution, the microhardness, and the tensile properties of tube samples were analyzed using tests and numerical simulations. The results show that the microstructure of each part of the welded joint was free of damaged features, such as a creep cavity and intergranular cracks. The microhardness of the weld was higher than that of the base metal. In the tensile test, the welded joints broke at the weld metal at room temperature and at the side of the TP304H base metal at a temperature of 550 °C. The tensile fracture morphology demonstrated a change from a ductile fracture to a hybrid fracture when the temperature rose. The fusion zone and base metal on the TP304H side were the stress concentration areas of the welded joint, which easily sprouted cracks. This study holds significant reference value in assessing the safety and reliability of dissimilar steel welded joints in superheater units.

## 1. Introduction

Dissimilar material welding is widely used in various fields because, in some cases, we need to use different types of materials and must join them together to form a complete structure. When conducting dissimilar material welding, the most common welding material used is dissimilar steel, and because of the different coefficients of thermal expansion, chemical compositions, physical properties, and other significant differences of the steel, the welded dissimilar steel is much more complex than when the same steel is welded. Problems associated with dissimilar steel welding, such as carbon migration, poor metallurgical bonding in the fusion zone, relatively complex stress fields at joints, microstructural deterioration, and the presence of cracks near the weld, severely affect the quality and safety of the welded joint [1,2,3,4]. TP304H and T22 are common dissimilar steel welding combinations in production. In thermal power generating units, many welded structures are manufactured using welded austenitic stainless-steel and low-alloy steel dissimilar metals [5,6,7]. Such welded joints operate under different temperatures and pressures for long periods, which necessitates the requirements of high temperature stability, high pressure bearing capacity, and chemical stability. The main problem associated with this kind of dissimilar steel welded joint is that the service life of the joint is shorter than the design life, and failure is mainly due to the low plastic cracking along the weld interface [2]. This kind of failure is usually sudden, and it easily causes damage to the welded structure, affecting normal production and resulting in huge economic losses. It is of great significance to study the microstructure and properties of dissimilar steel welded joints to ensure the safe and stable operation of units.

In the dissimilar steel welding process, because of changes to the metallurgical organization of the welded area or the generation of new metallurgical organizational arrangements, the performance of welded joints usually degrades. In order to evaluate the quality of welded joints, the microstructures of each zone of welded joints were obtained by optical and scanning electron microscopy, and the reasons for the different microstructures of dissimilar welded joints were studied [3]. Zhang [8] characterized semicircular and linear layered textures in fusion zone and tensile fracture features based on the microstructure. Ou [9] paid more attention to the evolutionary process of the microstructural morphology of the welded joint and obtained the structure–property relationship of the joint. Zhai [10] further investigated the effect of the microstructural evolution on the creep properties. Mittal [11] selected different combinations of welding processes and welding materials and evaluated the performance of the joint based on the microhardness and fracture results. The failure mode of most of the welded structures was brittle fracture under low stress levels, with little apparent plastic deformation before fracture. Battahar [12] found that the presence of macroscopic defects or the location of cracks in mechanical structures was the main source of fatigue crack initiation. Comprehensive studies by Wang [13] and Chen [14] showed that the mechanism of crack initiation and propagation exists mainly in the fusion line region, as shown in different microstructures in dissimilar welded joints. Sun [15] mainly investigated the effects of chemical composition and grain size on near-threshold fatigue crack growth behavior. In addition, some research focused on adjusting the strength–toughness matching of welded joints. Aghajani Derazkola [16] analyzed the formation mechanism of intermetallic compounds during dissimilar welding. Chen [17] increased the tensile strength of dissimilar welded joints by reducing the content of intermetallic compounds. Fan [2] analyzed failed dissimilar steel tube samples and found that the stress concentration effect was the main reason for the cracking of the dissimilar steel weld before the design life. For the study of the stress distribution pattern in welded joints, Zhang [18] obtained the residual stress distribution in adjacent welded joints through numerical simulation methods. Adomako [19] discussed the deformation mechanism of dissimilar welded joints and predicted the tensile behavior of the welded joint. Therefore, the early failure of welded joints can be avoided by analyzing the evolution of the microstructure and diffusion mechanism of the elements, paying attention to the location of the area of the stress concentration in the welded joints. The variation law of the temperature and stress field during the welding process can be obtained, and the residual stress and deformation behavior of welded joints can be predicted through the numerical simulation of welded joints. Sun [20] discussed the crack propagation path based on the extended finite element method. The results of the study confirmed the effectiveness of numerical simulation for predicting the expansion of crack emergence in welded joints. The feasibility of using numerical simulation to optimize the welding parameters and the reliability of the mechanical property analysis of welded joints based on numerical simulation results has been verified in the literature [21,22,23,24,25]. Therefore, based on previous studies, experimental and numerical simulation methods were used in this study for predicting mechanical properties across dissimilar material welded joints.

In this study, a welded joint of TP304H/T22 dissimilar steel, which has served for a long time at the outlet of a secondary superheater of a 350 MW subcritical boiler, was taken as the research object. The mechanical properties of the welded joint were analyzed. The microstructure, microhardness, and tensile properties of each region of the welded joint were studied using experiments. Additionally, the finite element method was utilized to analyze the high-temperature tensile process of the welded joint. According to the stress distribution law of the welded joint and the evolution of the microstructure of the welded joint, the weak areas of the welded joint were obtained. This study provides a reference for the reliability of dissimilar steel welded joints under long-term high-temperature and high-pressure operation and the avoidance of early failure phenomena such as cracking.

## 2. Experimental Materials and Methods

**Figure 1:**
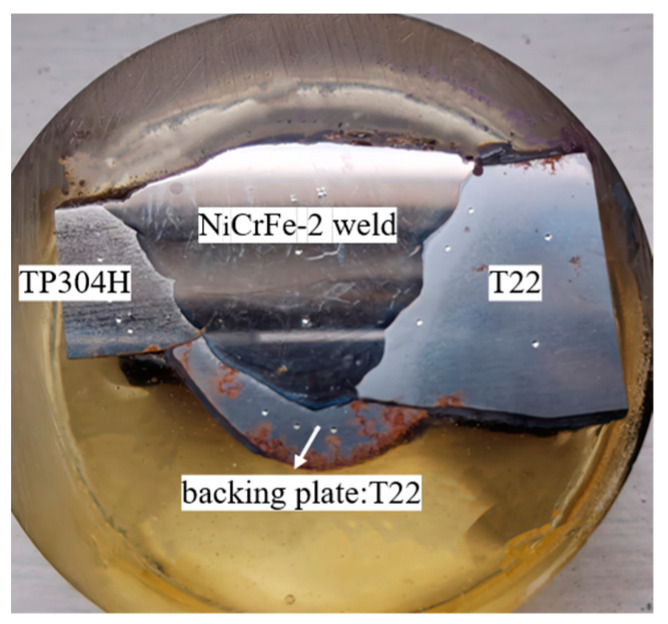
Section morphology of the welded joint.

**Figure 2:**
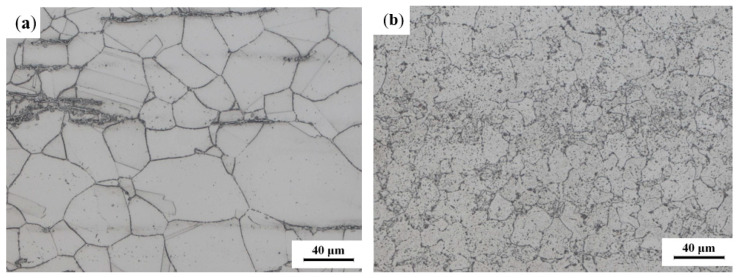
Microscopic morphology of the base metal: (**a**) TP304H; (**b**) T22.

The TP304H/T22 dissimilar steel welded joint of a 350 MW subcritical boiler was taken as the research object. The dissimilar steel welded joint was located on the roof of the outlet section of the boiler superheater, and it had been continuously operated under high-temperature and high-pressure conditions over a long period of time. The transverse cross-sectional morphology of the welded joint is shown in Figure 1. The TP304H and T22 tube samples had diameters of *Φ*50.8 × 7.4 mm and *Φ*50.8 × 12.2 mm, respectively. The chemical compositions are shown in Table 1 and Table 2. Because of the different wall thickness of the base metal on both sides, the dissimilar steel weld added a backing plate (T22). The contact position between the backing plate and the base metal on both sides was not welded together. A nickel-based alloy welding wire was selected as the welding material for the dissimilar steel joints. The welding wire contained high amounts of Ni and Cr elements and a certain amount of alloy elements, such as Nb, Mo, and Ti. Its linear expansion coefficient was between pearlitic steel and austenitic steel, which can reduce the generation of thermal stress, prevent the diffusion and migration of carbon near the fusion zone, and ensure the weldability and performance of the joint [5]. The metallographic structure of the welded joint of TP304H/T22 dissimilar steel was observed and analyzed with a ZEISS IMAGER A1m metallographic microscope, and the metallographic structure of the base metal is shown in Figure 2. It can be seen that the TP304H is a single austenite structure, and the T22 consists of ferrite, pearlite, and granular bainite with uniform distribution. The two dissimilar materials, TP304H and T22, are metallurgically compatible and easily form new phase structures or metal compounds during the welding process. They have a certain weldability and form good, welded joints.

The microstructure of the welded joint was observed using a Thermo Scientific Apreo field-emission scanning electron microscope, and the second phase composition was analyzed with X-ray energy-dispersive spectrometry (EDS). Specimens were prepared according to standard metallographic procedures for steel, including embedding, grinding, polishing, and solution etching, prior to observing the microstructure. A digital display Vickers hardness tester was used to measure the Vickers microhardness on the specimen surface at a fixed spacing of 1 mm. The rectangular tensile specimens with full wall thickness were prepared and tested using an electronic universal tensile testing machine at room temperature and 550 °C.

## 3. Microstructure of Welded Joints

**Figure 3:**
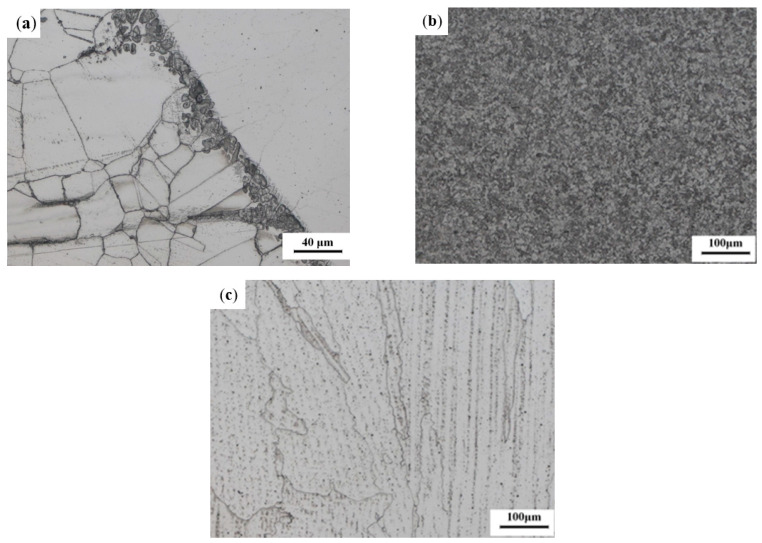
Microstructure of the welded joint: (**a**) TP304H side adjacent to the fusion line; (**b**) T22 side heat-affected zone; (**c**) weld.

In the dissimilar steel joint tube sample, a small amount of the precipitated phases and inclusions in the base metal zone on the TP304H side were observed. Unevenly sized equiaxial twins connected, and the austenite grains of the base metal at the melting boundary directly contacted the welded molten pool cell to generate epitaxial nucleation, as shown in Figure 3a. In addition, the homogenization caused by solid diffusion was small due to the matrix of the fusion zone on the TP304H side still being austenite and the structure being tightly arranged [9]. Close to the fusing line side, the austenite grains grew epitaxially from the base metal to the weld recrystallization zone.

The microstructure of the heat-affected zone on the T22 side is shown in Figure 3b. There were more evenly dispersed massive ferrite and a small amount of lathy ferrite. In this area, the bainite morphology essentially disappeared and dispersed carbides precipitated at the grain boundary. This was due to the existence of a large amount of chromium in the T22 matrix, which caused the carbide to form a grain structure at high temperature. Compared to the metallographic organization of the T22 base metal, the grain refinement in the heat-affected zone had a better ability to resist crystallization cracks. In addition, as the same material was used between the backing plate and the base metal, the welding and fusion performance improved, and no damage characteristics, such as creep cavity and intergranular cracks, were found, avoiding the fracture of joints caused by crystal cracks as the source of fatigue cracks.

Figure 3c shows the microstructure of the center area of the weld. The weld metal composition phase was mainly austenite with coarse grains, large spacing between dendrite arms and cell crystals, and small chrome-rich carbides precipitated at the grain boundary, among which some oxides were included to produce shrinkage holes [6].

**Figure 4:**
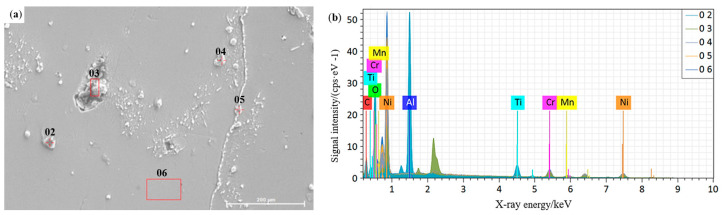
Weld precipitation phase: (**a**) location distribution of EDS measurement points; (**b**) EDS test results.

As shown in Figure 4a, the weld had white precipitates distributed in and at the grain boundary, which were analyzed using EDS. The EDS spectrum results are shown in Figure 4b. The mass fractions of the different elements are shown in Table 3. The elements mainly detected include Fe, Cr, and Nb. Nb easily combined with C and N to form the Nb (C, N) phase, which was diffusely distributed within the inner crystal and at grain boundaries [26]. This was beneficial for improving the strength and impact toughness of the welded joint. At the measurement point 02, the elements O, Al, and Ti were mainly detected. Refer to the research results of other similar literature, where the higher oxygen content in the weld metal could cause oxidative corrosion effects, which could lead to cracks in the joint during future service processes [27]. Combined with Figure 3c, the microstructure of the weld showed that the weld grain boundary precipitated tiny carbides, and there was a tendency to interconnect into a chain, and the second phase particles with small and many fine sizes inhibited the growth of grains by pinning grain boundaries [15], which reduced the size of previously generated austenite grains and easily induced cracks at the weld.

## 4. Mechanical Properties of Welded Joints

### 4.1. Hardness

**Figure 5:**
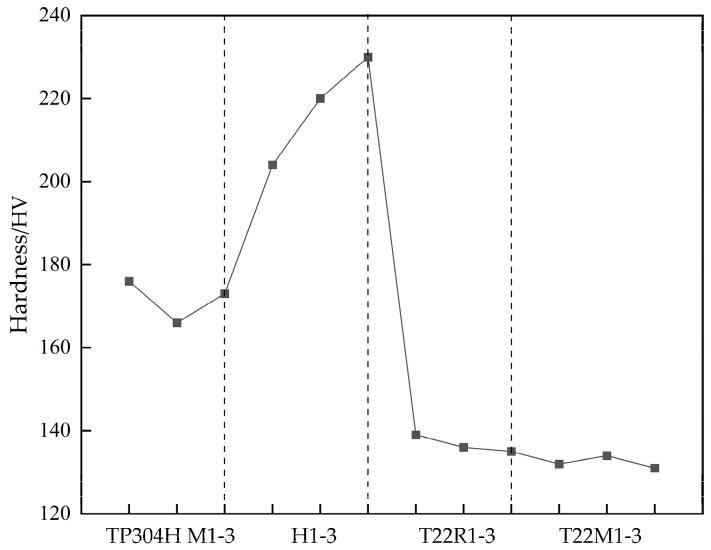
Vickers hardness distribution of the TP304H/T22 dissimilar welded joint.

The micro-Vickers hardness test was performed on the tube samples with a test load of 10 kgf and a holding time of 12 s. The tube sample was utilized for microhardness testing. The hardness value measurement numbering method started from the fusion boundary R onwards to the weld or the direction of the base metal. M was used to indicate the distribution of the measurement points on the base metal, H to indicate the measurement points on the weld, and R to indicate the measurement points in the heat-affected zone. The number represents the serial number of the measurement points, with each measurement point having an interval of 1 mm. The Vickers hardness value test results are shown in Figure 5. The average hardness of the TP304H base metal was 172 HV, and the average hardness of the T22 base metal was 132 HV. According to DL/T 438-2016 “*Technical Supervision Regulations for Metals in Thermal Power Plants*”, the hardness of TP304H is required to be 147–202 HV, and the hardness of T22 is required to be 131–189 HV. The weld zone was made of NiCrFe-2, which has good strength and plasticity at high temperature. The test results show that the hardness of the base metal on both the T22 and TP304H sides met the standards, with the average hardness of T22 being close to the lower limit of the standard and the average hardness of TP304H being moderate.

As shown in Figure 5, the hardness values of the specimen in the TP304H base metal zone were similar to those in the fusion line, indicating that no phenomena such as interdiffusion and new phase generation occurred between the two parts. The chemical composition, as shown in Table 2, and the microstructure, as shown in Figure 3a, further prove this conclusion. The average value of the hardness of the specimen in the TP304H base metal was lower than the average value of the hardness in the weld. The hardness of the specimen increased abruptly in the weld zone due to the hindrance of the dislocation slip of the grain boundary in the heat-affected zone on the TP304H side (Figure 3a). The difference in the hardness values between the weld and the base metal might lead to joint cracking. The heat-affected zone on the T22 side had higher hardness values than the T22 base metal, with an average hardness value of 137 HV. The metallographic organization of the heat-affected zone on the T22 side (Figure 3b) showed that the grain size in the heat-affected zone was smaller compared to the base metal, and the hardness at this location increased because of the fine grain strengthening effect. The maximum hardness of the welded heat-affected zone also reflects the strength of the welded heat-affected zone. The higher the strength, the poorer the toughness and plasticity, and the easier it is to produce or extend cracks [11]. The lower hardness value of the base metal on the T22 side indicates that there is some degree of damage to the pipe during use, and the decrease in the hardness value is mainly caused by carbide spheroidization [28].

### 4.2. Tensile Properties at Different Temperatures

**Figure 6:**
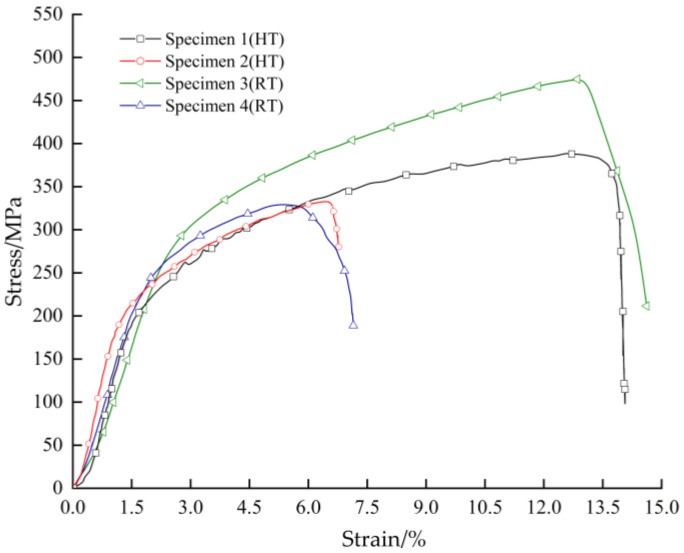
Tensile stress–strain curves of the TP304H/T22 dissimilar welded joint.

Four samples at the same position on the pipe sample were cut with the weld seam as the center to make full-thickness rectangular tensile samples, numbered 1, 2, 3, and 4. The tensile properties of the welded joints at room temperature (RT) and 550 °C are shown in Table 4, and the stress–strain curves are shown in Figure 6. Specimens 1 and 2 were high-temperature tensile specimens, and specimens 3 and 4 were room-temperature tensile specimens. The high-temperature tensile specimens were fractured at different locations on the TP304H side. Specimen 1 was fractured on the TP304H side of the base metal, indicating that the strength of the joint was higher than that of the carbon steel base metal. The tensile fracture morphology was rosy red, the fracture plane was at an angle of approximately 45° to the tensile axis, and the fracture was wedge shaped. Specimen 2 was broken along the fusion line on the TP304H side, and the fracture morphology was also rosy red. Bai conducted high-temperature tensile tests on unserved joints, and the results showed that all joints fractured on the low-alloy side [7]. In this study, high-temperature tensile tests were performed on welded joints after long-term service, which fractured on the austenite side. All room-temperature tensile specimens fractured at the weld on the TP304H side. The fracture morphology was gray–white, and the plastic deformation at the fracture site was large.

Compared with the fracture positions of the tensile test, the plastic deformation of the welded joints at 550 °C was significantly lower than the deformation at room temperature. This was because the dislocation slip of the grains was prone to occur at high temperatures. In addition to the cross-slip of the spiral dislocation, new dislocation was activated by stress, leading to dynamic recovery and softening [8]. The austenitic–low-alloy steel dissimilar welded joints were used as pressure-bearing parts of the thermal power generating units. At some interfaces and microcracks inside the crystal, thermal stress and tissue stress caused stress concentration, resulting in dislocations [4]. This effect became more obvious with the increase in tensile temperature, which led to the weakening of the strain strengthening effect, accelerating the uniform plastic deformation period, as well as reducing the uniform plastic deformation period. As a result, the specimen produced local plastic deformation earlier, that is, the phenomenon of “retraction neck”.

**Figure 7:**
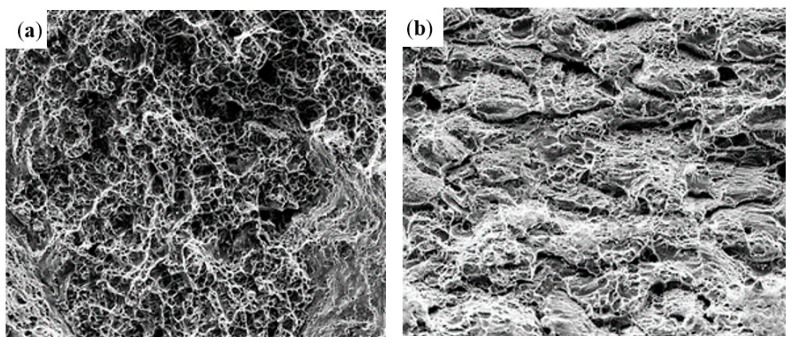
Tensile fracture microscopic morphology: (**a**) room temperature; (**b**) high temperature.

The tensile fracture microstructures of the joint specimens at different temperatures are shown in Figure 7. The fracture location of the room temperature tensile joint occurred in the center of the weld, and there was a large area of dimples on the fracture. These dimples were closely distributed and mostly equal axial or slightly elongated dimples (Figure 7a), showing typical ductile fracture characteristics. The high-temperature tensile fracture was mainly composed of dimples, cleavage planes, and slip. In the local part, when the external stress gradually increased, there was a “retraction neck” phenomenon. When it shrank to a certain extent, tearing occurred, resulting in the overall fracture of the weld and forming dimples [17]. Moreover, Figure 7b indicates that the fracture mechanism of the high-temperature tensile specimens was a mixed tough–brittle fracture.

## 5. Numerical Simulation of High-Temperature Tension of Welded Joints

**Figure 8:**
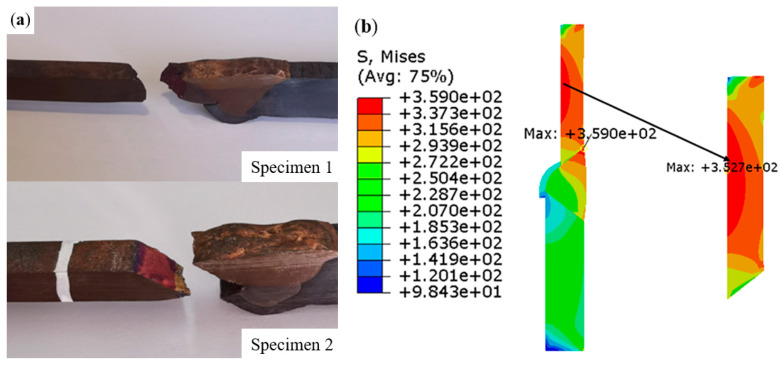
High-temperature tensile testing of the welded joints: (**a**) experiment; (**b**) simulation.

An axisymmetric finite element model of the welded joint was established for the numerical simulation analysis. The physical parameters of the TP304H/T22 dissimilar steel welded joints used for the numerical simulation are shown in Table 5. Mesh division was performed using CAX4R linear reduced integration elements. The finite element model comprised 3492 nodes and 3302 elements. To simulate the real unidirectional tensile test of the welded joint of the TP304H/T22 dissimilar steel at 550 °C, the temperature field was predefined in the tensile region of the welded joint model. One end of the welded joint model was constrained, and a displacement load was applied to the other end. Figure 8 demonstrates the results of the numerical simulation and the tensile test.

In actual working conditions, the superheater pipeline is mainly subjected to circumferential tension caused by high-temperature and high-pressure steam, as well as bending stress and axial tensile stress caused by secondary stress [2]. The stress concentration effect increases the local stress value, which is the weak position of the joint. The stress distribution of welded joints should be paid more attention. Based on the distribution law of equivalent stress in unidirectional tensile, the danger zone of a welded joint was defined to avoid cracking due to the stress concentration during service. In Figure 8b, two high stress zones can be observed in the TP304H base metal and the TP304H side weld fusion zone of the joint. According to the finite element simulation, the maximum equivalent stress concentrated on the base metal of TP304H at the initial stage of loading, and the effect of stress concentration played a dominant role. In the middle and late stages of loading, because of the different properties of the materials in each section, nonuniform deformation occurred in the tensile process, and the maximum equivalent stress appeared in the weld fusion zone of the TP304H side. The interaction between the different materials at the weld position was more obvious. In the actual damage process, the damage would preferentially appear in the two high stress zones and then evolve into microcracks, which together with the macroscopic crack propagation would lead to the fracture of the specimen, as shown in Figure 8a.

## 6. Conclusions

In this study, the microscopic morphology, microhardness, and tensile properties of TP304H/T22-type dissimilar steel welded joints in long-term service conditions were analyzed, and the following conclusions were obtained:

(1) No damage features, such as creep cavities and intergranular cracks, were found in the microstructure of the welded joints. On the side of the TP304H close to the fusion line, austenite grains grew from the base metal matrix to the weld recrystallization zone. The microstructure of the heat-affected zone (HAZ) on the T22 side was refined, which provided the HAZ with better crack initiation resistance.
(2) The microhardness test results show that tube sample weld zone and base metal had a significant hardness difference. The microstructure analysis revealed that the strong resistance of the grains to dislocation movement during deformation and the significant increase in dislocation proliferation resulted in the highest hardness values at the weld and that crack initiation and propagation were more likely to occur.
(3) The high-temperature tensile test of the welded joint showed that fracture occurs in the base metal, or the TP304H side fusion zone. In addition, the numerical simulation results also show that in the actual high-temperature tensile process, in those two parts of the high stress area, damage defects were prone to initiation. The high-temperature tensile fracture morphology indicates that the fracture form was a mixed fracture. The joint fractured at the weld under room-temperature tensile tests, and the fracture showed the characteristics of a ductile fracture.
(4) The tensile test results showed that the welded joints fractured at different locations after a long period of service than those unserved. Combined with the numerical simulation results, the weak position of the welded joints of the in-service dissimilar steel was clarified. This is a reference for the safe and stable operation of in-service dissimilar steel welded joints.

## Figures and Tables

**Table 1:** Chemical compositions of T22 (mass fraction, %).

Material	Cr	Mo	Mn	Si	C	S	P	Fe	Ni	Nb
T22	2.08	0.92	0.44	0.22	0.088	0.016	0.0098	Balance	-	-

**Table 2:** Chemical compositions of TP304H (mass fraction, %).

Material	Cr	Ni	Mn	Si	C	P	S	Fe	Mo	Nb
TP304H	18.82	9.82	1.52	0.55	0.045	0.026	0.012	Balance	-	-

**Table 3:** Normalized mass percentages of elements at different measurement point locations (%).

Locations	C	O	Al	Si	Ti	Cr	Mn	Fe	Ni	Nb	Hg
02	9.55	34.97	26.94	-	18.38	2.22	1.58	-	6.35	-	-
03	7.33	-	-	2.34	2.69	15.41	3.31	10.43	26.25	32.23	-
04	5.52	-	-	0.98	-	16.51	4.45	18.51	50.66	3.37	-
05	9.03	-	-	0.51	-	14.23	3.72	17.26	53.86	1.40	-
06	2.75	-	-	0.49	-	16.30	2.29	18.32	58.53	-	1.32

**Table 4:** Tensile properties of the TP304H/T22 welded joint specimens.

Specimen	Tensile Strength (MPa)	Standard Tensile Strength (GB/T 5310-2017) (MPa)	Fracture Positions
1 (HT)	389	≥381 (TP304H at high temperature)	TP304H side base metal
2 (HT)	334		TP304H side fusion line
3 (RT)	476	≥515 (TP304H at room temperature)	Welding seam
4 (RT)	330	≥415 (T22 at room temperature)	Welding seam

**Table 5:** Physical properties of the TP304H/T22 welded joint at 550 °C.

Material	Density (t/mm^3^)	Expansion (°C^−1^)	Poisson Ratio	Young’s Modulus (MPa)	Yield Stress (MPa)	Plastic Strain
T22	7.8 × 10^−9^	1.46 × 10^−5^	0.3	169,000	105	0
					210	0.02
TP304H	7.8 × 10^−9^	1.86 × 10^−5^	0.31	156,000	159	0
					320	0.02
N06600	7.8 × 10^−9^	1.5 × 10^−5^	0.31	182,000	240	0
					560	0.02

## Data Availability

Not applicable.
